# Minimally Invasive Plate Osteosynthesis for Proximal Humerus Fractures: A Retrospective Study Describing Principles and Advantages of the Technique

**DOI:** 10.1155/2018/5904028

**Published:** 2018-06-03

**Authors:** Riccardo Luigi Alberio, Matteo Del Re, Federico Alberto Grassi

**Affiliations:** Department of Orthopedics and Traumatology, University of East Piedmont, Hospital “Maggiore della Carità”, Via Mazzini 18, Novara, Italy

## Abstract

**Background:**

The aim of this study was to evaluate the clinical and radiographic results after minimally invasive plate osteosynthesis (MIPO) for proximal humerus fractures. Potential advantages of this approach include the easier exposure of the greater tuberosity and the limited surgical dissection around the fracture site.

**Materials and Methods:**

From October 2011 to March 2016, thirty-nine patients (32 women, 7 men) with a mean age of 64.9 years (range: 48–80) were surgically treated with the MIPO technique for proximal humeral fractures. According to Neer classification, there were 12 two-part, 24 three-part, and 2 four-part fractures and 1 two-part fracture-dislocation; the AO/OTA system was also used to categorize the fractures. The Constant-Murley (CMS) and the Oxford Shoulder (OSS) Scores were used to evaluate shoulder function.

**Results:**

Thirty-four patients were available for clinical and radiographic evaluation at a mean follow-up of 31.8 months (range: 12–54 months). All fractures healed and no postoperative complications occurred. Full recovery of pretrauma activities was reported by 27 patients, while 7 patients presented mild functional limitations. The mean absolute CMS was 75.2 (range: 55–95), the mean normalized CMS was 90.5 (range: 69–107), and the mean OSS was 43.7 (range: 31–48). The only statistically significant correlation was found between the female gender and lower absolute CMS and OSS. Radiographic evaluation revealed varus malunion in 4 cases and valgus malunion in 1 case, while incomplete greater tuberosity reduction was detected in 4 cases. All malunions were related to inadequate reduction at time of surgery and not to secondary displacement.

**Conclusions:**

MIPO for proximal humeral fractures is an effective and safe surgical procedure. The limited tissue dissection allows minimizing the incidence of nonunion, avascular necrosis, and infection. The technique is not easy, requires experience to achieve mastery, and should be reserved for selected fracture patterns. In our experience, the main advantage of this approach consists in the direct access to the greater tuberosity, thus facilitating its anatomic reduction and fixation.

## 1. Introduction

The incidence of proximal humerus fractures is increasing for two main reasons that reflect the bimodal distribution of these injuries. On one side, progressive aging of the population is associated with a rise in low energy injuries, particularly among women over 60 with osteopenia. On the other side, the wide participation in sport activities and the reduced mortality in traffic accidents are related to a growing rate of high-energy fractures requiring treatment [1, 2].

Owing to the wide variety of anatomo-clinical conditions, treatment options for proximal humeral fractures vary considerably. Surgical treatment is usually performed for displaced and/or unstable fractures with the goal of restoring normal anatomy as well as allowing early shoulder rehabilitation. Several procedures can be adopted for fracture fixation, but plating remains the most popular method of osteosynthesis. Undoubtfully, the introduction of locking plates has led to a great improvement in clinical outcomes and has widened indications for internal fixation, but the rate of failures and complications is still considerable.

While for fractures of the surgical neck many surgeons prefer nailing to plating, three- and four-part fractures are mainly treated with locking plates. Even in severe fracture patterns, plating is widely performed and this trend has reduced the rate of anatomic shoulder replacement performed for fractures [3, 4].

Open reduction and internal fixation through the deltopectoral approach may require extensive soft tissues dissection and strong deltoid retraction, particularly for gaining adequate access to the greater tuberosity [5, 6]. To overcome these drawbacks, a minimally invasive, deltoid-splitting approach has been adopted for plating proximal humeral fractures.

The minimally invasive plate osteosynthesis (MIPO) technique facilitates the exposure of the greater tuberosity and allows respecting vascularity and soft tissues around the fracture, thus preserving better conditions for bone healing [7–9]. However, this approach exposes the patient to an increased risk of axillary nerve injury if compared to the traditional deltopectoral approach [10, 11].

In this retrospective study, we report the clinical experience achieved in a consecutive series of 39 patients treated with MIPO technique for proximal humeral fracture.

## 2. Materials and Methods

From October 2011 to March 2016, thirty-nine patients were surgically treated with the MIPO technique for displaced proximal humerus fractures by a single surgeon at a single institution. In this clinical series, neither a treatment protocol nor inclusion/exclusion criteria were preventively established. The MIPO technique was performed for fractures in which reduction and plating had reasonable chances of success without exposing the anteromedial aspect of the proximal humerus. Fractures with wide diastasis between fragments, high-energy injuries with relevant soft tissue interposition between bone fragments, four-part fractures (excluding valgus impacted), and articular (head-splitting or severely impacted) fractures were not considered suitable for the MIPO technique. These lesions were either plated through a deltopectoral approach or treated with shoulder arthroplasty.

This series included 32 women and 7 men, with a mean age of 64.9 years (range: 48 to 80 years) at the time of injury. The dominant arm was involved in 24 cases (61.5%).

The mechanism of injury was a fall from standing height in 21 patients (53.8%), a traffic accident in 10 patients (25.6%), a sport accident in 5 patients (12.8%), and a fall from great height in 3 patients (7.7%).

All the patients underwent radiographic examination of the shoulder before surgery and a CT-scan of the shoulder was performed in 14 patients (35.9%) to better define the fracture pattern. According to Neer's criteria, the series included 12 two-part, 24 three-part, and 2 four-part fractures and 1 two-part fracture-dislocation. Fractures were also classified according to the AO/OTA system, as reported in Table 1.

Time span between trauma and surgery averaged 7,25 days, with a range of 2 to 17 days.

At the latest follow-up, clinical evaluation of the patients was performed using the absolute and normalized Constant-Murley Score (CMS) [12, 13] and the Oxford Shoulder Score (OSS) [14]. Radiograms of the shoulder (A-P view in internal and external rotation) were also taken to detect avascular necrosis (AVN), nonunion, malunion, implant malposition, and screws perforation.

Mann–Whitney U test (SPSS 10.0) was used to compare functional outcomes with nonparametric statistical variables. P values less than 0,05 were considered statistically significant.

### 2.1. Surgical Technique

All patients are operated under general anesthesia in the beach-chair position. The affected limb overhangs the edge of the table and the patient's head is firmly secured with tape. The C-arm image intensifier is placed on the opposite side in order to visualize the fractured humerus with minimal obstacle for the surgeon.

**Figure 1:**
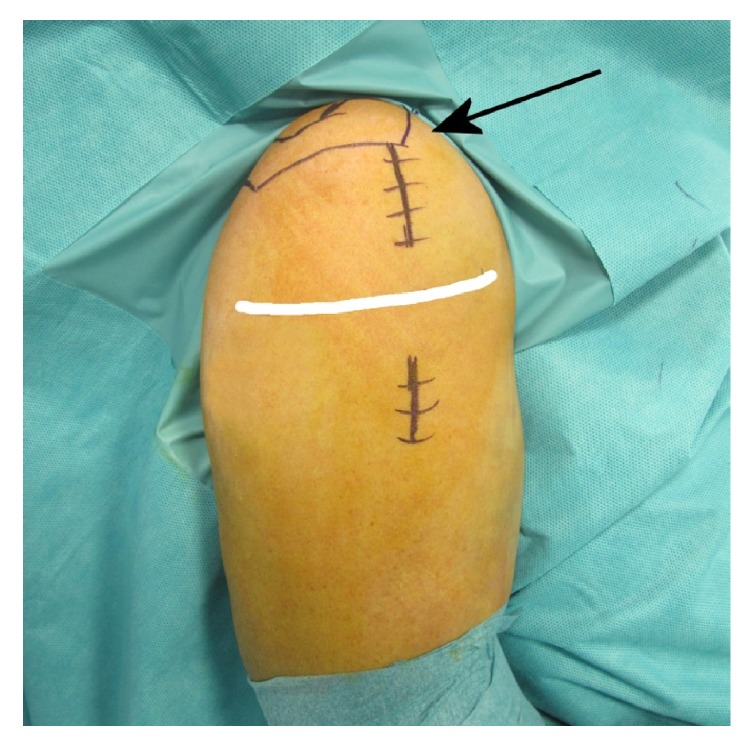
Surgical field of a right shoulder showing the two skin incisions performed for the MIPO technique. The proximal incision starts at the anterolateral corner of the acromion (arrow) and is approximately 5 cm long; the length of the distal incision varies according to the number of diaphyseal screws implanted. The white line shows the level and course of the axillary nerve.

Reduction of surgical neck fractures is attempted by closed manipulation before starting the procedure. The skin incision begins at the anterolateral corner of the acromion and extends 5 cm distally in line with the proximal humerus (Figure 1). The deltoid muscle is split along the anterior rafe and the subacromial bursa is removed. The cord-like axillary nerve is palpated on the deep surface of the deltoid approximately 2-3 cm below the inferior end of the wound.

Strong nonabsorbable sutures are placed in the rotator cuff at the bone-tendon junction for facilitating humeral head and tuberosities reduction. One or two 2 mm K-wires can be inserted in the humeral head and used as joysticks for helping fragment realignment. Head reduction can be maintained by inserting two additional K-wires in a position not interfering with plate implantation.

Using the insertion handle, the plate (Philos, DePuy Synthes, Solothurn, Switzerland) is slid along the humeral surface under the deltoid muscle and the axillary nerve. The plate should be seated just lateral to the bicipital grove and kept in contact with bone. In case of three- or four-part fractures, the greater tuberosity must be previously reduced and laid under the plate. In its final position, the superior edge of the plate should be 1 cm below the top of the greater tuberosity to avoid subacromial impingement.

A second skin incision is started at the level of the first diaphyseal screw hole of the insertion handle and extended distally according to the number of diaphyseal screws needed (Figure 1). After having aligned the inferior end of the plate with the humeral shaft, temporary fixation of the plate is performed by inserting two 1.6 mm K-wires in the humeral head through holes in the aiming device.

**Figure 2:**
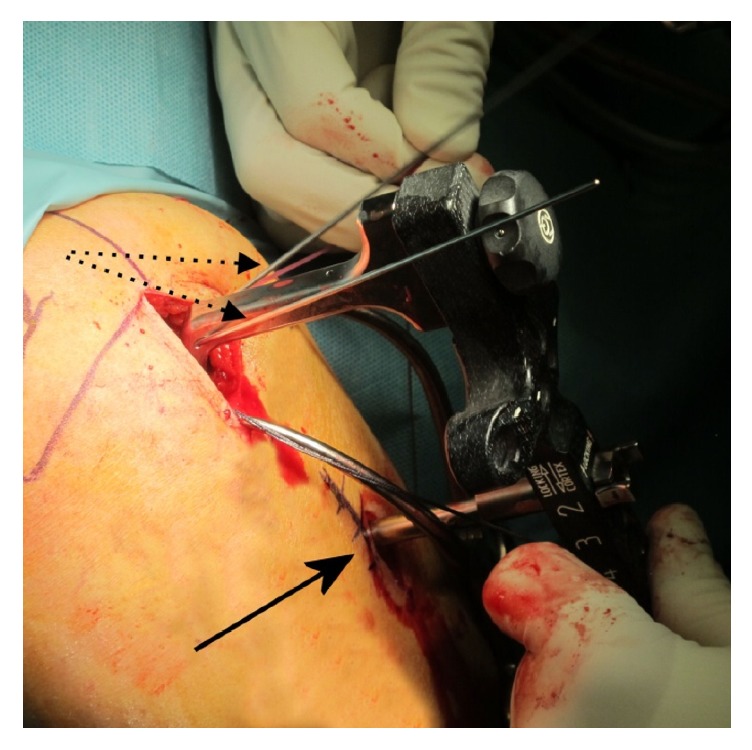
Insertion handle of the Philos plate with two 1.6 mm K-wires (dotted arrows) inserted in the humeral head for temporary fixation of the proximal fragment(s). Sleeve system (arrow) for insertion of bicortical locking or nonlocking screws in the humeral shaft.

The reduction is checked with the image intensifier and a nonlocking bicortical screw is inserted in the humeral shaft using the sleeve system of the insertion handle (Figure 2). This step allows improving the alignment of the shaft with the head by taking advantage of the shape of the plate.

After having inserted four angular stable screws in the humeral head and one or two angular stable screws in the shaft, the insertion handle is removed. Additional screws can be inserted in the humeral head by abducting the arm in order to gain access to the inferior epiphyseal holes of the plate without jeopardizing the axillary nerve.

Fixation can be augmented by passing strong nonabsorbable sutures through the rotator cuff and anchoring them into the peripheral holes of the plate.

After wound closure, the arm is placed in a sling. Assisted passive elevation of the shoulder to 90° (hand to the top of the head) in the supine position is immediately started. The patient can also mobilize the upper limb gently for personal care and clothing as pain permits.

If X-rays taken at three weeks do not show any worrisome finding, the patient is encouraged to perform light activities of daily living and active elevation of the shoulder without resistance. The sling is gradually abandoned, but it is recommended to wear it for protection when out in public.

Further clinical and radiographic evaluations are carried out six weeks and three months after surgery. During this period, stretching and strengthening exercises are performed under the supervision of a physiotherapist to gradually improve shoulder function.

## 3. Results

Thirty-four patients were available for clinical and radiographic evaluation at a mean follow-up of 31.8 months (range: 12 to 54 months). Four patients declined to show up for logistical problems, while one patient was untraceable. All dropouts (12.8%) were women; therefore the last follow-up population included 27 female and 7 male patients with a mean age of 64.8 years (range: 48 to 80 years).

**Figure 3:**
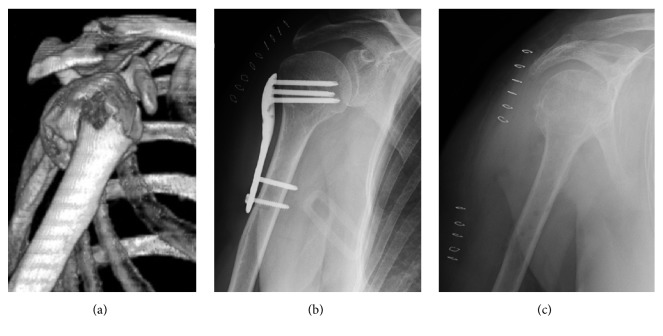
(a) Two-part varus nonimpacted fracture of the surgical neck (AO/OTA type A3) in a 57-year-old woman. (b) Postoperative radiogram: the plate was not flush with the bone surface and caused painful impingement during rehabilitation. (c) Three months after fixation the plate was removed; the fracture healed uneventfully.

No intraoperative complications occurred. In one patient, hardware removal had to be performed three months after fixation, because the plate was not adherent to the bone surface and caused subacromial impingement during rehabilitation (Figure 3). The fracture healed uneventfully without secondary displacement.

At follow-up, the mean absolute CMS was 75.2 (range: 55-95) and the normalized one was 90.5 (range: 69-107) (Table 2). Pain was absent in 23 patients (67.6%), mild in 10 (29.4%), and moderate in one patient (3%).

Complete return to preinjury activity level was reported by 27 patients (79.4%), while 7 patients (20.6%) had some degree of functional impairment.

Active ROM showed no restriction in 19 patients (55.9%). Mild, moderate, and severe loss of shoulder motion were observed in 9 (26.5%), 3 (8.8%), and 3 (8.8%) patients, respectively.

The mean OSS was 43.7 (range: 31 to 48). In 30 patients (88.2%) the score was higher than 40, indicating an excellent subjective outcome, and in 3 patients (8.8%) it ranged from 35 to 40, revealing a mild subjective functional disability (Table 2).

**Figure 4:**
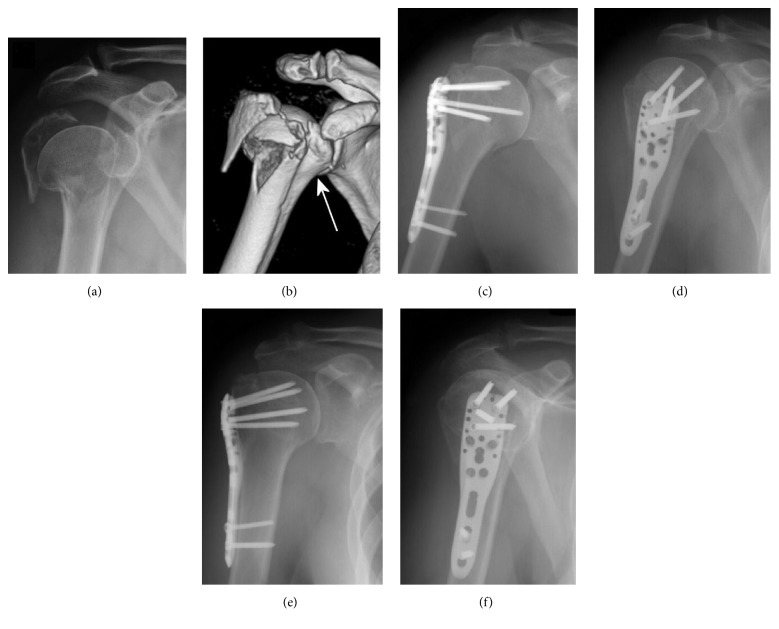
(a)-(b) Three-part valgus impacted fracture with severe displacement of the articular segment in a 48-year-old woman (AO/OTA type C2); the lesser tuberosity was in continuity with the shaft (arrow). (c)-(d) Postoperative radiograms in external (c) and internal (d) rotation showing anatomical reduction of the fracture. (e)-(f) The same radiographic views three months after surgery demonstrating union with no secondary displacement.

**Figure 5:**
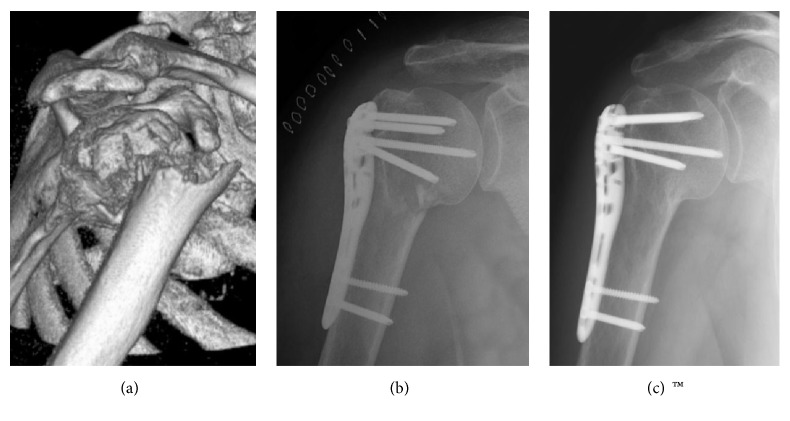
(a) Two-part varus nonimpacted fracture of the surgical neck (AO/OTA type A3) in a 71-year-old woman. (b) Anatomical reduction was not achieved at time of surgery: postoperative radiogram showed mild varus angulation of the humeral head. (c) One year after surgery fracture was healed without any aggravation of the varus.

Radiographic evaluation at follow-up did not show any case of AVN, nonunion, or screw perforation (Figure 4). Mild to moderate varus malunion of the humeral head was observed in 4 (11.8%) patients and valgus malunion in one (3%). Nonanatomical reduction of the greater tuberosity was evident in 4 (11.8%) patients. All malunions were not subsequent to secondary displacement, but were related to incomplete fracture reduction at the time of surgery (Figure 5).

Statistical analysis revealed a significant correlation between female gender and worse absolute CMS and OSS (Table 3). Functional scores did not correlate either with age (cut-off 70 years) or with fracture pattern. Worse outcomes were observed in patients with malunion, but differences in CMS and OSS with the rest of the patients were not statistically significant (Table 4).

## 4. Discussion

Progressive aging of population is the most important factor contributing to the increasing incidence of proximal humeral fractures in highly developed countries [1, 2]. Most of these lesions can be successfully treated by conservative means, but in many cases surgical treatment is required and several options are available.

Internal fixation with locking plates is widely performed, but the rate of complications and revisions is still high. Two different meta-analyses showed that the incidence of complications after this procedure is around 45% and that more than 13% of the patients undergo reoperation for different reasons, with the most common being loss of reduction and screw perforation [5, 15].

When plating a fracture of the proximal humerus, there are two conflicting goals to pursue: the first one is to achieve an anatomical reduction and a stable fixation of the bone fragments; the second one is to minimize surgical damage on soft tissues and vascularization. In fact, poor reduction and inadequate fixation almost inevitably lead to shoulder function with unsatisfactory clinical results. Conversely, extensive surgical dissections can compromise the healing process, increasing the risk of avascular necrosis (AVN), nonunion, loss of reduction, screw perforation, and infection.

The MIPO technique has been adopted to overcome these challenges in some fracture patterns. The transdeltoid approach facilitates exposure of the greater tuberosity without the need for retracting vigorously the deltoid or violating the rotator cuff. Therefore, the fracture site can be adequately exposed and reduction can be achieved with minimal impairment of the healing process.

The distal extension of the anterosuperior approach is insidious, specifically if compared to the deltopectoral access. No patient of this series required an uninterrupted incision to expose the upper part of the humerus or an inferior exposure to the humeral shaft. However, some authors recommend an extended transdeltoid approach for plating proximal humeral fractures. Extreme care and attention must be paid to isolate and protect the axillary nerve at the level of the mid-deltoid. In our opinion, the extended deltoid-splitting approach might be adopted as backup plan in case of need, but should be avoided whenever possible.

Since limited exposure of the metaphyseal region is achieved with the MIPO technique, it is preferable to perform the surgical procedure within few days from injury. This is because the formation of early fibrous callus in the proximal humerus might hinder fragment reduction. In this series of patients, the average time span between trauma and surgery was one week, an adequate interval to perform MIPO without encountering obstacles to reduction caused by the interposition of organized fibrous tissue. When surgery is delayed for more than two weeks, this potential problem should be taken into account, particularly when a relevant gap between fragments is present at the metadiaphyseal level.

Studies comparing the minimally invasive approach with the traditional deltopectoral approach reported better functional outcomes, lower surgical time, lower blood loss, shorter hospital stay, and faster fracture healing for the former [9, 16–18].

Numerous studies reported clinical outcomes and complications of the MIPO technique for fractures of the proximal humerus [19–27]. The average absolute CMS of these studies is 75.1 points, a value that matches the average score of 75.2 measured in our series of patients.  Table 5 summarizes the most relevant data reported in previous studies dealing with the MIPO technique.

In this series of patients, a significant difference in clinical results was detected according to gender: women showed worse outcomes than men not only in terms of absolute CMS, as could be expected, but also with regard to the OSS. However, it must be highlighted that this difference might be biased by the small sample size and the numerical disparity among the two groups (27 women versus 7 men).

The MIPO technique should not be considered an easy procedure and the surgeon must pay particular attention to avoid axillary nerve injuries. The use of the insertion handle might be helpful for this purpose, since this instrument facilitates sliding of the plate on the bone surface. No iatrogenic nerve injuries occurred in this series of patients, but other authors reported an incidence of this severe complication between 3,1% and 4,7% [19, 20, 25, 26].

The minimally invasive approach has the advantage of respecting indirect bone healing, thanks to a surgical exposure aimed at preserving fracture haematoma. This condition accelerates the healing process and allows an early removal of the plate when needed. In this clinical series, one patient required plate removal three months after fixation because of plate malpositioning and the fracture healed uneventfully. Other authors reported higher percentage of hardware removal for the same reason [11, 24], highlighting the importance of precise plate positioning to prevent subacromial impingement.

No secondary displacements were observed in the postoperative period and screws position was not a critical factor for maintaining reduction achieved at time of surgery. Even though several authors highlighted the importance of placing an inferior screw to support the medial calcar, this aspect does not seem essential with the MIPO technique. The handle of this specific system does not have a hole for the insertion of an oblique calcar screw, in order to avoid the risk of iatrogenic damage to the axillary nerve.

In our opinion, the short time required for fracture healing is the main factor that decreases the risk of fixation failure, even when anatomical reduction is not achieved. In case of osteoporotic bone, adequate calcar support might also be accomplished by some degree of shortening that can provide contact and impaction between head and shaft. Metaphyseal shortening should not exceed 2 cm; otherwise tuberosity reduction might be troublesome.

**Figure 6:**
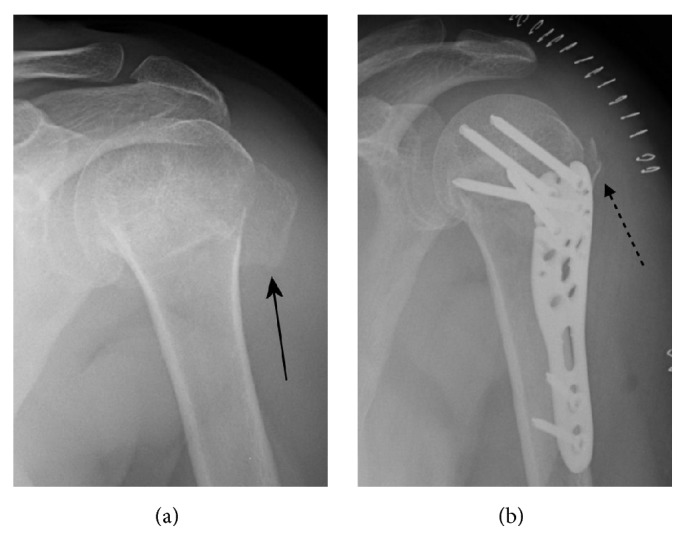
(a) Three-part varus impacted fracture with posterior displacement of the greater tuberosity (arrow) in a 55-year-old woman (AO/OTA type B1). (b) Nonanatomical reduction of the greater tuberosity (dotted arrow). The distortion of the normal bone morphology was minimal and did not influence the clinical outcome.

Malunion is the most frequent complication reported in literature [21, 23, 24] as well as in this study. It is mainly due to incomplete fracture reduction at the time of fixation, but it may also result from secondary displacement of bone fragments in the postoperative period, particularly in osteoporotic bone. In our experience, all malunions were caused by inadequate fracture reduction at the time of surgery. Varus malunion of the humeral head occurred in 11.8% of the patients and correlated with worse clinical outcomes, even though the difference was not statistically significant. It has been stated that only varus angulations greater than 20° correlate with poor clinical outcomes [22]. Malunion (nonanatomical reduction) of the greater tuberosity was observed in 11.8% of the patients as well, but the distortion of normal anatomy was negligible and did not compromise the clinical results (Figure 6).

The absence of AVN is consistent with the results of other studies [20–23] and the highest reported incidence of this complication after the MIPO technique is 8.2% [19]. Fixation through the deltopectoral approach is associated with higher rates of AVN [5, 15], but it should be underlined that the occurrence of this complication is greatly conditioned by the fracture type. Even though the minimum follow-up in this study is one year, it is still too early to exclude the risk of this complication for some patients conclusively.

Screw perforation can be caused by inaccurate length measurements during surgery, but it can occur later as a consequence of fixation failure, humeral head collapse, or tuberosity resorption. In literature, high rates of screws perforation have been reported by many authors [19, 25–27], but no case was observed in this study. The MIPO technique might be helpful in containing the risk of screw perforation, but prevention relies primarily on a careful selection of patients and a meticulous surgical procedure with optimal fluoroscopic intraoperative control.

The limitations of this study include the retrospective design and the limited number of patients. Moreover, inclusion criteria were not based on specific fracture patterns; the MIPO technique was indicated and performed by a single experienced shoulder surgeon, whose subjective evaluation of the lesion was critical in decision making. Current classification systems of proximal humeral fractures do not provide significant discrimination criteria for choosing between plating with MIPO or standard open technique. However, some fractures were not considered suitable for MIPO because it was felt that the limited surgical exposure could not allow adequate reduction or fixation. In our opinion, the MIPO technique should not be performed in case of wide diastasis between fragments, particularly after high-energy trauma, in presence of a “free floating” (not impacted) head fragment and in fractures where there might be the necessity to move from fixation to shoulder replacement intraoperatively.

Several options are now available for the surgical management of proximal humeral fractures, ranging from percutaneous pinning to reverse shoulder arthroplasty, but currently available literature cannot be used to derive any standardized, evidence-based treatment algorithm [28]. Significant variation in surgical practice is often indicative of a lack of scientific data and consensus in the medical community regarding optimal treatment.

The MIPO technique allows to achieve satisfactory clinical outcomes with a low complication rate, but success of treatment is also influenced by the individual level of surgical expertise. The procedure is not easy and the learning curve is clearly steeper for low-volume trauma surgeons and for severe fracture patterns. Minimal exposure of the fracture site, respect of the rotator cuff, easy access to the greater tuberosity, and reliable fixation allowing early rehabilitation are some of the advantages worth mentioning. The MIPO technique should be indicated after an accurate evaluation of the lesion by means of reproducible, high quality imaging studies. If plating is the selected treatment option and the fracture pattern requires a wide exposure for reduction, or if the surgeon does not feel confident with this approach, then the deltopectoral approach should be preferred.

## Figures and Tables

**Table 1:** Classification of the fractures according to AO/OTA system.

**AO/OTA**		**n**
**A**	**1**	1
	**2**	0
	**3**	4

**B**	**1**	15
	**2**	15
	**3**	0

**C**	**1**	3
	**2**	1
	**3**	0

**Table 2:** Clinical data and outcome scores of patients evaluated at follow-up.

	Gender/Age	Fracture Type		F/U (months)	Malunion	Constant Score		Oxford Shoulder Score
		*Neer*	*AO/OTA*			*Absolute*	*Normalized*	
**1**	F/75	3-p	B2	54		85	107	48
**2**	F/55	3-p	B1	52	Tuberosity	83	100	45
**3**	F/77	3-p	B2	51		62	78	37
**4**	M/53	3-p	B2	50		89	98	47
**5**	F/60	3-p	B1	49		83	100	48
**6**	M/59	4-p	C1	49	Tuberosity	81	90	47
**7**	F/64	2-p	B2	47		66	81	41
**8**	F/57	2-p	A3	47		67	80	41
**9**	F/57	3-p	B1	44	Tuberosity	80	96	48
**10**	F/77	3-p	C1	43		59	74	40
**11**	F/55	3-p	B1	39	Tuberosity	78	93	37
**12**	F/69	2-p	A3	39		73	90	48
**13**	M/71	3-p	B1	37		72	83	45
**14**	M/52	2-p disl.	A1	37		95	105	48
**15**	M/62	2-p	A3	33		91	104	48
**16**	F/80	2-p	B2	32		77	97	43
**17**	F/77	3-p	B2	30	Varus	64	81	42
**18**	M/69	2-p	B2	29		83	95	48
**19**	F/66	3-p	B2	27		66	81	45
**20**	F/73	2-p	B2	26	Valgus	55	69	40
**21**	F/71	2-p	A3	25	Varus	67	84	31
**22**	F/50	3-p	B2	24		73	86	45
**23**	M/73	3-p	B1	23		87	101	48
**24**	F/52	2-p	B2	23		76	91	39
**25**	F/70	2-p	B1	23		59	72	41
**26**	F/75	3-p	B1	22		79	100	48
**27**	F/72	3-p	B2	21	Varus	77	97	44
**28**	F/71	2-p	B2	19		83	105	47
**29**	F/69	3-p	B1	16		77	95	42
**30**	F/72	3-p	B1	15	Varus	59	74	43
**31**	F/64	3-p	B1	15		72	88	40
**32**	F/49	2-p	B2	15		78	92	42
**33**	F/48	3-p	C2	13		85	101	44
**34**	F/60	3-p	B1	12		77	92	47

						**75.2**	**90.5**	**43.7**

**Table 3:** Correlation between clinical scores and gender.

	**Constant Score**		**Oxford Shoulder Score**
	***Absolute***	***Normalized***	
**Female** *n* = 27	72,59	89,04	42,81
**Male** *n* = 7	85,43	96,57	47,29
	***p = 0,003*** ^*∗*^	*p = 0,089*	***p = 0,006*** ^*∗*^

**Table 4:** Correlation between clinical scores and malunion.

	**Constant Score**		**Oxford Shoulder Score**
	***Absolute***	***Normalized***	
**No malunion** *n* = 25	76,56	91,84	44,4
**Malunion** *n* = 9	71,56	87,11	41,89
	*p = 0,312*	*p = 0,284*	*p = 0,218*

**Table 5:** Comparison of clinical results and complications reported with the MIPO technique.

Authors	Patients (*n*)	Mean age (years)	F/U (months)	Implant	Absolute Constant Score	Complications and failures
*Acklin et al.* (2013)	124	62	18	Philos	75	4 injuries of the ventral branch of axillary nerve (without clinical implications) 8 AVN 7 screw perforation

*Jung et al.* (2013)	38	72.4	18	Philos	75.7	1 injury of axillary nerve (axonotmesis) 1 fixation failure 1 plate impingement 1 superficial wound infection

*Sohn et al.* (2014)	62	57	37	Philos	80 (2-part) 74 (3-part) 62 (4-part)	3 plate impingement 1 screw perforation 5 varus malunion 1 greater tuberosity migration

*Koljonen et al.* (2015)	40	63	17.8	Philos	75	1 fixation failure with screws perforation 1 varus malunion

*Chen et al.* (2015)	27	67.3	20.8	Philos	89.4	1 screw perforation

*Falez et al.* (2015)	76	68.5	12	Philos	71	7 plate impingement 5 varus malunion 3 screw perforation 1 AVN 2 greater tuberosity resorption 1 greater tuberosity migration

*Gönç et al.* (2016)	31	58.4	12	Philos	73.2	2 temporary nerve injury (1 axillary, 1 radial) 1 deep infection 3 screw perforation 1 AVN 2 varus malunion

*Park et al.* (2014)	21	61	20.8	Periarticular PH-LP	79.4 (6 months) 82.7 (1 year)	1 delayed union 1 axillary nerve injury 1 screw perforation

*Barco et al.* (2012)	23	62	36	NCB-PH	64	2 deep infection 3 malunion (2 varus, 1 greater tuberosity) 3 screw perforation

**Average**	49.1	63.5	21.4		75.1	

**Current study**	34	64.8	31.8	Philos	75.2	9 malunion (4 varus, 1 valgus, 4 greater tuberosity) 1 plate impingement
